# Hopeine—(Alkaloid from Hops)

**Published:** 1885-12-20

**Authors:** 


### HOPEINE—(ALKALOID FROM HOPS.)

This alkaloid, which appears to be different from the lupuline of Griessmeyer, is said to be most abundant in the American hop. It occurs as a white crystaline powder, or in the form of needles a third of an inch long. It is very sparingly soluble in water, but dissolves freely in alcohol, the solution having the most intense bitter taste and a pronounced smell of hops. Chemically, it bears a close resemblance to morphine. In its physiological action it is a pure narcotic, even fatal doses producing no irritant effect; but it contracts the pupil, raises the temperature and increases the frequency of the pulse at first, but afterwards diminishes it. The deep sleep which it induces is apt to be preceded and followed by peculiar hazy hallucinations. It has been used as a hypnotic in doses ranging from one-third to six-tenths of a grain for adults, though three-quarters of a grain has produced symptoms of poisoning. The dose does not have to be increased on account of the system becoming habituated to the drug. The "toxic dose" is not much above a grain and a half for adults, and not over nine-tenths of a grain for children.—*New York Medical Journal.*

